# A novel cancer vaccine with the ability to simultaneously produce anti-PD-1 antibody and GM-CSF in cancer cells and enhance Th1-biased antitumor immunity

**DOI:** 10.1038/sigtrans.2016.25

**Published:** 2016-11-18

**Authors:** Hongwei Tian, Gang Shi, Qin Wang, Yiming Li, Qianmei Yang, Chunlei Li, Guoyou Yang, Min Wu, Qian Xie, Shuang Zhang, Yang Yang, Rong Xiang, Dechao Yu, Yuquan Wei, Hongxin Deng

**Affiliations:** 1 State Key Laboratory of Biotherapy/Collaborative Innovation Center for Biotherapy, West China Hospital, Sichuan University, Chengdu, Sichuan, China; 2 Department of Biomedical Sciences, University of North Dakota, Grand Forks, North Dakota, USA; 3 Department of General Medicine, West China Hospital, Sichuan University, Chengdu, Sichuan, China; 4 Department of Immunology, Nankai University School of Medicine, Tianjin, China

## Abstract

Tumor escape from immune-mediated destruction has been associated with immunosuppressive mechanisms that inhibit T-cell activation. A promising strategy for cancer immunotherapy is to disrupt key pathways regulating immune tolerance, such as program death-1 (PD-1/PD-L1) pathway in the tumor environment. However, the determinants of response to anti-PD-1 monoclonal antibodies (mAbs) treatment remain incompletely understood. In murine models, PD-1 blockade alone fails to induce effective immune responses to poorly immunogenic tumors, but is successful when combined with additional interventions, such as cancer vaccines. Novel cancer vaccines combined with antibody may offer promising control of cancer development and progression. In this investigation, we generated a novel tumor cell vaccine simultaneously expressing anti-PD-1 mAbs and granulocyte-macrophage colony stimulating factor (GM-CSF) in CT26 colon cancer and B16-F10 melanoma. The antitumor effect of the vaccine was verified by therapeutic and adoptive animal experiments *in vivo*. The antitumor mechanism was analyzed using Flow cytometry, Elispot and *in vivo* intervention approaches. The results showed that tumor cell vaccine secreting PD-1 neutralizing antibodies and GM-CSF induced remarkable antitumor immune effects and prolonged the survival of tumor-bearing animals compared with animals treated with either PD-1 mAbs or GM-CSF alone. Antitumor effects and prolonged survival correlated with strong antigen-specific T-cell responses by analyzing CD11c^+^CD86^+^ DC, CD11b^+^F4/80^+^ MΦ cells, increased ratio of Teff/Treg in the tumor microenvironment, and higher secretion levels of Th1 proinflammatory cytokines in serum. Furthermore, the results of ELISPOT and *in vivo* blocking strategies further confirmed that the antitumor immune response is acquired by CD4 and CD8 T immune responses, primarily dependent on CD4 Th1 immune response, not NK innate immune response. The combination of PD-1 blockade with GM-CSF secretion potency creates a novel tumor cell vaccine immunotherapy, affording significantly improved antitumor responses by releasing the state of immunosuppressive microenvironment and augmenting the tumor-reactive T-cell responses.

## Introduction

A major obstacle in tumor cell vaccine technology is inefficient stimulation of an immune response to induce antitumor effects. The effective immune response that leads to meaningful antitumor effects requires not only an increase in immune activation, but also reduction of suppressive or inhibitory elements of the immune system.^1–3^ To generate efficient antitumor immune responses while maintaining self-tolerance, host reactions are tightly regulated through a combination of stimulatory and inhibitory signals. The combination of PD-1 blockade with GM-CSF production in a single cell may be an effective approach to improve the anti-tumor response.

PD-1, an immunoinhibitory receptor belonging to the CD28 family,^4,5^ is predominantly expressed on activated T cells.^6^ In addition, PD-1 is found on antigen-specific T cells that are chronically exposed to antigen.^1,7,8^ Recent studies have documented a critical role in T-cell regulation involving PD-1 and its ligands, PD-L1 (B7H1) and PD-L2 (B7-DC). PD-L1 is expressed on hematopoietic cells and can be upregulated on activation.^9^ Tumor cells that express PD-L1 using this pathway as a mechanism to evade recognition/destruction by the immune system.^10–13^ PD-L2 expression is restricted only to macrophages and dendritic cells and is also up-regulated by activation.^9^ Suppression of effector T-cell function by PD-1 engagement induces deletion and apoptosis,^14,15^ inhibition of proliferation and production of cytokine such as interleukin (IL)-2 and IFN-γ, and together with chronic antigen exposure, results in T-cell exhaustion.^1^

Granulocyte-macrophage colony stimulating factor (GM-CSF) is a potent cytokine activator of antigen presenting cells (APCs) and has an important role in breaking tolerance and the development of antitumor immune response.^16^ It is regarded to be ideal adjuvant owing to its potent activation of dendritic cells (DCs) and myeloid progenitor maturation. GM-CSF secreting cancer vaccines have been reported to induce massive accumulation of DCs at the inoculated site and in turn to activate tumor specific T cells to induce an anti-tumor response.^17,18,19,20,21^

In our study, we co-expressed anti-PD-1 mAbs and GM-CSF in poorly immunogenic B16-F10 melanoma cells and the immunogenic CT26 colon carcinoma cells. We first generated the tumor cell vaccine that can secret bioactive antibodies and cytokines simultaneously and verify its anti-tumor effects *in vivo*. The results showed that the tumor cell vaccine secreting PD-1 neutralizing antibodies and GM-CSF induced remarkable antitumor immune effect after immunization in mice. Adoptive animal experiments confirmed that the immune effect induced was specificity. The mechanism showed that the combined vaccine could raise CD11c^+^CD86^+^ DC, CD11b^+^F4/80^+^ MΦ cells and the ratio of Teff/Treg in the tumor microenvironment. Blocking experiments *in vivo* further confirmed that the antitumor immune response is acquired by CD4 and CD8 T immune responses, mainly dependent on CD4 Th1 immune response, not NK innate immune response. In brief, our research supplied a novel vaccine design strategies in cancer immunotherapy.

## Materials and methods

### Cell and animals

The mouse colon carcinoma cell line CT26 and mouse skin melanoma B16-F10 were purchased from American Type Culture Collection (ATCC). The cells were cultured in Dulbecco’s modified Eagle’s medium (DMEM, Gibco, NewYork, NY, USA) and RPMI-1640 medium (Gibco) supplemented with 10% heat-inactivated fetal bovine serum, 100 U ml^−1^ penicillin and 100 mg ml^−1^ streptomycin (Sigma-Aldrich) at 37 °C in a humidified atmosphere containing 5% CO_2_, respectively. Female 4- to 6-week-old C57BL/6 and Balb/c mice were obtained from the laboratory Animal Center of Sichuan University, Chengdu, China. All animal experiments were carried out in accordance with standard guidelines and approved by the Animal Care Center in State Key Laboratory of Biotherapy, West China Hospital, Sichuan University.

### Vaccine preparation

The complete mouse mAb specific for PD-1 (patent No: US 20030026800A1) and mouse GM-SCF (NM_009969.4) was subcloned into the lentivirus vector and packaged into recombinant lentivirus particles using a standard procedure. CT26 colon carcinoma and B16-F10 melanoma cell lines were infected with lentivirus particles co-expressing anti-PD-1 mAbs and GM-CSF cytokine, respectively. The stable CT26 and B16-F10 were selected by puromycin and developed into different clones such as C26 or B16-F10-PD-1 antibody alone, GM-CSF alone and PD-1 antibody with GM-CSF. These clones were irradiated with a sublethal dose X-ray (100 Gy)^22^ by irradiator (RS-2000 biological irradiator, Rad Source Technologies, Inc., Suwanee, GA, USA) and made into vaccines used for further study including detection of cytokine levels before or after irradiation and animal experiments *in vivo*.

### Bioactivity of PD-1 antibody analysis and cytokine evaluation

*In vitro,* the supernatants of irradiated groups expressing anti-PD-1 mAbs were collected. CD8^+^PD-1^+^ tumor-infiltrating lymphocytes (TIL) cells in CT26 Tumor were sorted by FACS (Flurescence-activated cell sorting) and labeled with CSFE (Carboxyfluorescein succinimudyl ester). Then the CD8^+^PD-1^+^ TIL was treated by anti-PD-1 mAbs in supernatant or commercial anti-PD-1 mAbs (1 μg ml^−1^) separately and cultured with PMA (12-O-Tetradecanoylophorbol 13-acetate) and ionomycin for 48 h. We analyzed the proliferation by FACS and measure interferon (INF)-γ, tumor necrosis factor (TNF)-α, IL-2 in supernatant by enzyme-linked immunosorbent assay (ELISA) to verify the bioactivity of anti-PD-1 mAbs. For *in vivo* cytokine analysis, mice were immunized with various tumor cell vaccines subcutaneously. Serum from each group including non-immunized group was collected through caudal vein on 1, 3, 5 and 7 day, respectively. Th1/Th2 cytokine and chemokine were analyzed by MilliplexMAP mouse cytokine/chemokine CBA kit (Millipore, Billerica, MA, USA).

### Therapeutic immunotherapy *in vivo*

For therapeutic vaccination, animals were implanted subcutaneously with 1×10^6^ B16-F10 melanoma cell or CT26 colon carcinoma on the right flank. At 3, 6 and 9 days, respectively, after tumor inoculation, the vaccines with anti-PD-1 mAbs and GM-CSF were immunized subcutaneously into the left flank of mice.^23^ Each mouse was immunized subcutaneously with 1×10^6^ B16-F10 melanoma cell or CT26 vaccine. Non-immunized group as control was injected with serum free medium alone. About 3 days after inoculation, the tumor volume was measured for six times every 2 days using the formula area=length×width and the survival curve was surveyed.

### Adoptive immunotherapy *in vivo*

As the method described in therapeutic immunotherapy, splenic lymphocytes of all groups were isolated by lymphocyte separation fluid (Tianjin Chuanye biochemical products company, Tianjin, China) according to the manufacturer’s standard procedure after the third immunization. Splenic lymphocytes were then counted and injected intravenously (1×10^7^cells per mouse) into mice which were challenged B16-F10 or CT26 tumor cells (1×10^6^ cells per mouse) subcutaneously. Adoptive immunotherapy of splenic lymphocytes was conducted on 3 day, 5 day, 7 day, 9 day after tumor challenge. About 1 week, tumor could be measured for six times every 2 days using the formula area=length×width and the survival curve could be surveyed.

### Immune cell subsets blocking assay *in vivo*

For depletion of immune cell subsets in *vivo*, mice were immunized as the schedule described in therapeutic immunotherapy and then injected intravenously with 100 μg anti-CD4 mAbs (eBioscience, San Diego, CA, USA), anti-CD8 mAbs (eBioscience), anti-NK mAbs (eBioscience) and isotype control rat IgG (eBioscience) at 1 day before tumor challenge and 6 day, 13 day after tumor challenge respectively. Tumor growth in different groups was measured^24^ for six times every 2 days using the formula area=length×width and the survival curve could be surveyed.

### ELISPOT assay *in vitro*

For analyzing the percentage of CD4^+^IFN-γ^+^and CD8^+^IFN-γ^+^ T cell in spleen at 5 days after immunization with vaccines, splenic cells were collected and plated (100 μl per well) in triplicate 1×10^5^ cells per ml, then cultured for 72 h in a CO_2_ incubator at 37 °C. To stimulate the secretion of IFN-γ, splenic cell of all groups were incubated in ELISPOT plates (R&D Systems, Inc., Minneapolis, MN, USA) in the presence of 0.5 μg ml^−1^ ionomycin mixed with 50 ng ml^−1^ PMA. These concentrations of mitogens were proved optimal to induce maximum stimulation without causing cytotoxic effects. Plates were detected with BCIP/NBT substrate and spots were quantified using a QuantiHub Elispot reader (MVS Pacific, LLC). The activity of IFN-γ-secreting cells was measured by the counting spots formed by IFN-γ secreted from individual cells and designated as spot-forming cells (SFCs). The number of SFCs was calculated by dividing the number of spots by the number of plated cells per well.

### Immunofluorescence analysis of T-lymphocyte infiltration in tumor tissue

After the last treatment of therapeutic immunotherapy, tumors in different groups were resected and cryo-cut for frozen sections. At the time of staining, slides were thawed, fixed in 4% PFA (Paraformaldehyde) for 10 min and subsequently washed in TBS with 0.1% tween (TBST) for 5 min three separate times. Then the slides were treated with 0.01% Triton-X 100 for 10 min and washed with TBST. Slides were also blocked with 10% goat serum in phosphate-buffered saline (PBS) for 30 min at room temperature. Primary rat anti-mouse CD8 (Abcam, Waltham, MA, USA), rat anti-mouse CD4 antibodies (Abcam) were incubated at 1:100 dilution for 30 min and then washed with PBST for 5 min three separate times. The secondary goat anti-rat IgG-Texas Red (ThermoFisher, Waltham, MA, USA) was used at 1:500 dilution for 30 min. Slides were washed with TBST for 10 min and mounted with DAPI. Pictures were taken with confocal laser scanning microscope under ×200magnification.

### Statistical analysis

Statistical analysis was performed by Graphpad Prism6 software (La Jolla, CA, USA). Statistical significance of difference between the two groups was determined by the paired-*T* test. The Kaplan–Meier plot for survival was assessed for significance using the log-rank test (SPSS software; version 16.0; SPSS Inc, Chicago, IL, USA). *P*<0.05 was considered significant.

## Results

### PD-L1 is highly expressed on tumor cell lines and tissues

**Figure 1:**
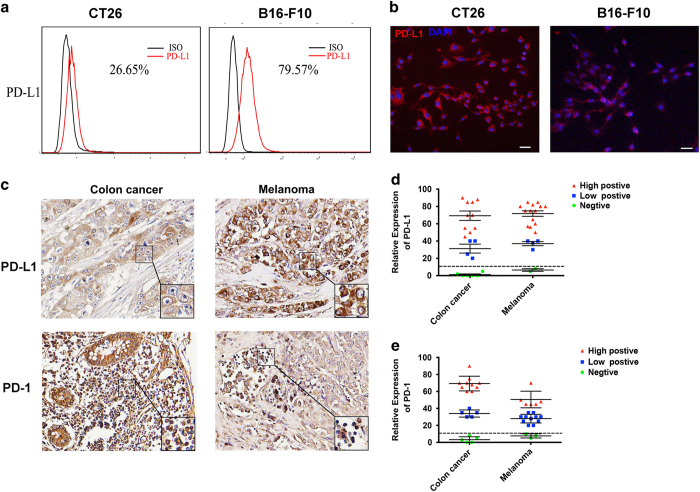
Expression of PD-L1 and PD-1 in mouse tumor cells and human tissues. (**a**) PD-L1 expression on the surface of CT26 and B16-F10 tumor cells was detected by FACS. The black and red line represents isotype and PD-L1, respectively. (**b**) Immunofluorescence analysis of PD-L1 expression on CT26 and B16-F10 tumor cells. Scale bar, 50 μm. Nuclei and PD-L1 showed blue and red, respectively. (**c**) TMA (Tissue microarrays) analysis for PD-1 and PD-L1 expression in colon cancer and melanoma tissues by immunochemistry. *n*=20. Scale bar, 50 μm. (**d**) TMA staining results for PD-L1 were assessed by their grades (high positive, low positive and negative). Each tumor contains 20 cases. Statistical charts were shown in different colors. (**e**) TMA staining results for PD-1 were assessed by their grades (high positive, low positive and negative). Each tumor contains 20 cases. Statistical charts were shown in different colors.

To analyze whether PD-L1 is highly expressed on different kinds of tumor cell line and tissue, and PD-1 is expressed on TIL in tumor microenvironment. We detected the PD-L1 expression on CT26, B16-F10 tumor cell lines by flow cytometry (Figure 1a) and immunofluorescence (Figure 1b), respectively. The results showed that PD-L1 percentage is CT26 (26.65%) and B16-F10 (79.57%). Immunohistochemistry analysis also revealed that approximately 80% of PD-L1 expression on tumor tissue and PD-1 expression on TIL were positive (Figure 1c) in clinical tissue samples include colon cancer, melanoma. The statistical graphs of PD-L1 and PD-1 expression are separately presented in Figure 1d and e. Taken together, PD-L1 is highly expressed either on tumor cell lines or cancer tissues. This may be the leading cause in inducing T-cell exhaustion in tumor microenvironment.

### PD-1^+^ TIL with low proliferation and cytokine secretion characteristics showed high positive percentage in tumor microenvironment

To investigate the percentage and characteristics of PD-1^+^ TIL in tumor microenvironment, CT26, B16-F10 tumor tissues were made into single cell suspension and stained with CD45-APC, CD4-FITC, CD8-PE/Cy7, PD-1-PE, CD44-PE/Cy5.5 and CD62L-APC/Cy7 flow antibodies to analyze the percentage of CD4^+^PD-1^+^ and CD8^+^PD-1^+^ TIL. The results showed that PD-1 was high positively expressed on TIL (Supplementary Figure 1a) both on CD4 and CD8 positive T cell in CT26 and B16-F10 models. To further explore the proliferation and cytokine secretion of PD-1^+^ TIL, especially in CD8^+^PD-1^+^ TIL, we sorted CD8^+^PD-1^−^ and CD8^+^PD-1^+^ TIL separately in a CT26 model and labeled them with CFSE *in vitro*. After PMA and Ionomycin stimulation for 72 h, the proliferation was detected by FACS and the results showed that there was no significantly difference in CD8^+^PD-1^+^ TIL with or without PMA and Ionomycin stimulation, but an obvious difference in CD8^+^PD-1^−^ TIL (Supplementary Figure 1b). CD4^+^PD-1^+^ TIL was also similarly analyzed (data not shown). Meanwhile, we collect the supernatant after 72 h in all groups and detect IFN-γ, TNF-α and IL-2 secretion by ELISA, it revealed a significant difference in IFN-γ, but no difference in TNF-α and IL-2 secretion (Supplementary Figure 1c).

### Tumor cell vaccine co-expressing PD-1 antibody and GM-CSF showed effective biological activity *in vitro*

**Figure 2:**
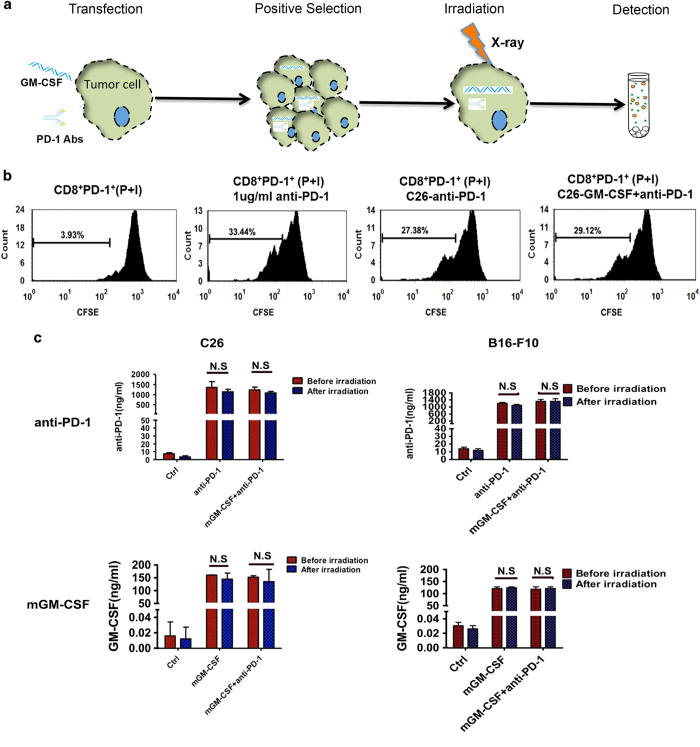
Preparation of cancer cell vaccine and biological activity analysis of PD-1 antibodies *in vitro.* (**a**) The schedule for vaccine preparation as shown. (**b**) CD8^+^PD-1^−^ and CD8^+^PD-1^+^ TIL in CT26 tumor were sorted and labeled with CFSE *in vitro*, then treated with supernatant from the CT-26-anti-PD-1 vaccine. The PD-1 antibody was used as a positive control at the concentration of 1 μg ml^−1^. After PMA and Ionomycin stimulation for 72 h, the proliferation was detected by FACS. (**c**) Supernatant of CT26 or B16-F10 expression of blocking PD-1 antibody and GM-CSF cytokine was collected before or after irradiation *in vitro*. The PD-1 antibody and GM-CSF expression were detected by Elisa. Data are means±s.d. (*n*=3) and are representative of three experiments (NS, no significance).

In order to assess the prepared tumor cell vaccines are in accordance with the optimized condition ‘no tumorigenicity but secreting cytokines’, we collected the cell supernatants after 48 h from cell vaccine irradiated with a sublethal dose X-ray and detected expression of PD-1 antibody and GM-CSF by ELISA, the schedule for vaccine preparation as shown in (Figure 2a). To ascertain whether tumor cell vaccine expressing PD-1 antibody still showed high biological activity after irradiation, we collected the supernatants of irradiated group and sorted CD8^+^PD-1^−^ and CD8^+^PD-1^+^ TIL cells separately in CT26 tumor, labeled them with CFSE *in vitro*. After PMA and Ionomycin stimulation for 72 h, the proliferation was detected by FACS. The commercial PD-1 antibody was used as a positive control at the concentration of 1 μg ml^−1^ (Figure 2b). We also detected the PD-1 antibody expression before or after irradiation. The results showed that expression of PD-1 antibody in CT26 and B16-F10 vaccine is 1232.65 and 1356.99 ng ml^−1^ separately after irradiation. The GM-CSF is 116.32 and 102.79 ng ml^−1^ separately. There are no significant differences between irradiation and non-irradiation groups (*P*>0.05) (Figure 2c). Also, we analyzed IFN-γ, TNF-α and IL-2 secretion by ELISA. Compared with controls, tumor cell vaccines co-expression anti-PD-1 mAbs and GM-CSF not only release the inhibition state of PD-1^+^ TIL in tumor microenvironment and promote the proliferation but also facilitate IFN-γ, TNF-α and IL-2 secretion of TIL (data not shown).

### Tumor cell vaccine co-expressing PD-1 antibody and GM-CSF exhibited enhanced antitumor effects for therapeutic immunotherapy *in vivo*

**Figure 3:**
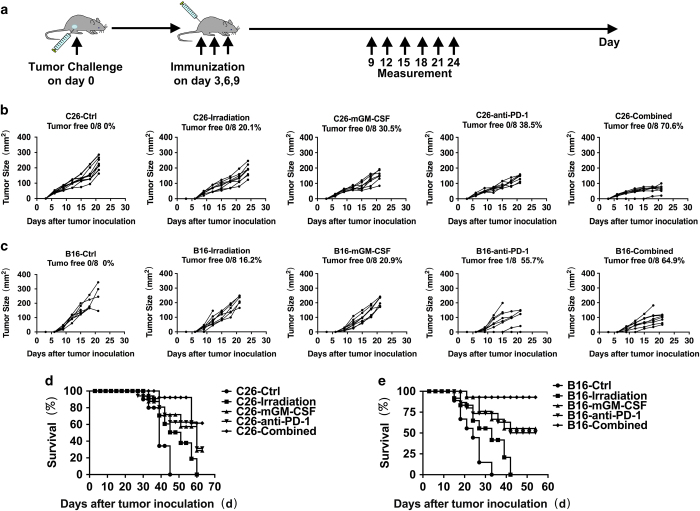
Therapeutic animal experiments *in vivo*. (**a**) The protocol of therapeutic immunotherapy. (**b**) 6-week-old Balb/c challenged with CT26 tumor (*n*=8), were immunized with vaccines expressing PD-1 antibody and GM-CSF cytokine as the protocol in (**a**) Tumor growth curve of C26 therapeutic animal model is also shown. Data are representative of three experiments. (**c**) 6-week-old C57BL/6 mice challenged with B16-F10 tumor (*n*=8), were immunized with vaccines expressing PD-1 antibody and GM-CSF cytokine as the schedule in (**a**) Tumor growth curve of B16-F10 therapeutic animal model is also shown. Data are representative of three experiments. (*n*=8). (**d**) Overall survival. Mice (*n*=8) were injected s.c. (subcutaneously) with 1×10^6^ CT26 tumor cells on day 0 and subsequently vaccinated and treated with vaccines as indicated in (**c**). Survival curve as shown and results are representative of three independent experiments. (**e**) Overall survival. Mice (*n*=8) were injected s.c with 1×10^6^ B16-F10 tumor cells on day 0 and subsequently vaccinated and treated with vaccines as indicated in (**d**). Survival curve as shown and results are representative of three independent experiments.

To verify whether the combined cancer vaccine co-expressing anti-PD-1 mAbs and GM-CSF can induce tumor growth inhibition *in vivo*, we formulated a therapeutic regimen (Figure 3a) for CT26 and B16-F10 tumor model separately. We strictly immunized mice in 3, 6 and 9 day separately after inoculated tumor cells and monitored tumor volume and survival. The combined group showed an average tumor area 66.39±23.5 mm^2^ (*P*<0.01) and inhibition rate 70.6% compared with control in a CT-26 model (Figure 3b). In a B16-F10 model, it also exhibited significant antitumor effects in an average tumor area of 91.01±26.54 mm^2^ (*P*<0.01) and 64.9% inhibition rate compared with controls (Figure 3c). Furthermore, we monitored the survival of all the groups, the results showed higher survival rates of the combined group compared with controls (*P*<0.01), the survival rate is 92.3% and 61.5% in a CT26 model at 45 days and 60 days, respectively (Figure 3d). Similarly, mice in a B16-F10 control group all died at 33 days after the beginning of inoculation, the survival rate of combined group is still 92.8% compared with controls at 33 days (Figure 3e). Taken together, these results suggest that combined vaccine co-expressing anti-PD-1 mAbs and GM-CSF significantly enhanced antitumor efficacy and improved survival compared with control group either in CT26 or B16-F10 tumor models.

### Combined vaccine-induced tumor-specific antitumor effects in adoptive immunotherapy *in vivo*

**Figure 4:**
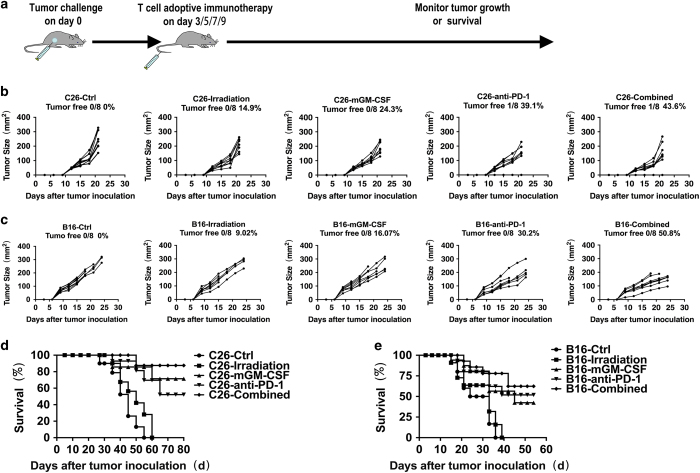
Adoptive animal experiments *in vivo*. Splenic lymphocytes of all groups were isolated after the third immunization and injected i.v. (intravenously; 1×10^7^ cells per mouse) into mice challenged with tumor. (**a**) Scheme of adoptive immunotherapy. (**b**) Tumor growth curve of C26 adoptive immunotherapy. Six-week-old C57BL/6 mice challenged with CT26 tumor (*n*=8), adoptive immunotherapy as (**a**). Data are representative of three experiments. (**c**) Tumor growth curve of B16-F10 adoptive immunotherapy (*n*=8). Data are representative of three experiments. (**d**) Kaplan–Meier survival analysis of C26 adoptive immunotherapy (*n*=8). Results are representative of three independent experiments. (**e**) Kaplan–Meier survival analysis of B16-F10 adoptive immunotherapy (*n*=8). Results are representative of three independent experiments.

To determine whether therapeutic immunotherapy produced tumor-specific antitumor effects *in vivo*, mice were immunized as described in the Materials amd Methods section (therapeutic immunotherapy). Splenocytes were isolated and used for adoptive immunotherapy as the planned scheme (Figure 4a). As expected, adoptive immunotherapy exhibited strong tumor-specific antitumor effects both in CT26 and B16-F10 models. The combined and control group in a CT26 model showed an average tumor area of 131.18±80.77 mm^2^ and 232.48±62.98 mm^3^, respectively. Compared with control, the combined group showed significant inhibition rate of 43.6% (*P*=0.003; Figure 4b). In a B16-F10 model, the average tumor area in combined and control groups is 149.59±28.56 mm^2^ and 304.21±50.13 mm^2^, respectively. The tumor inhibition rate reached 50.8% compared with control group (*P*<0.01; Figure 4c). Furthermore, the survival of combined group mice was markedly prolonged either in CT26 or B16-F10 models when compared to the control group (*P*<0.01). The survival rate of combined group mice in CT26 reached 87.5% at 55 days when all of the control group mice died (Figure 4d). In a B16-F10 model, the rate is 77.9% at 36 days (Figure 4e). Altogether, these results suggested that adoptive immunotherapy-induced tumor-specific antitumor effects.

### Combined vaccine upregulated the ratio of Teff/Treg and expression of Th1 cytokines in tumor microenvironment

**Figure 5:**
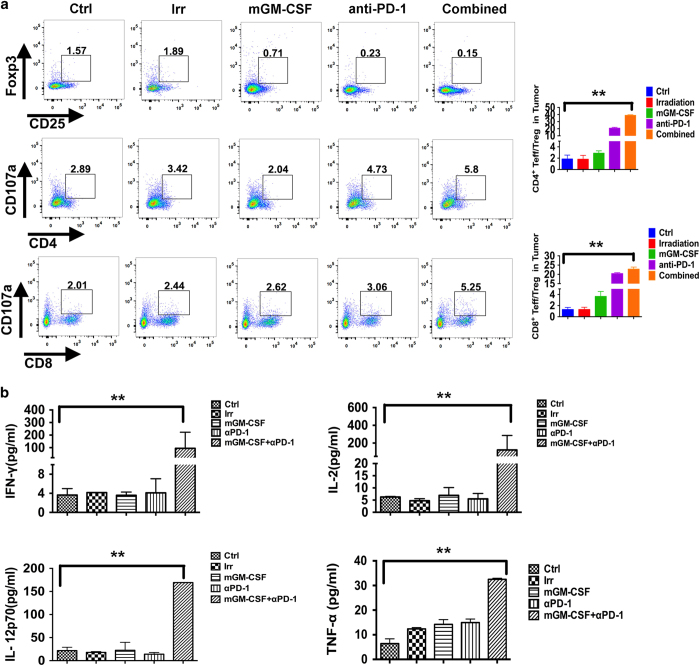
Variation of Teff/Treg in tumor microenvironment and cytokines in blood. The serum, spleen, lymph nodes and tumor samples in all groups were collected at 1, 3, 5 and 7days following the last immunization, respectively. The population changes of CD11c^+^CD86^+^DC, CD11b^+^Gr-1^+^MDSC, CD11b^+^F4/80^+^MΦ, Teff/Treg in spleen and tumor were analyzed by FACS. (**a**) Changes of Teff/Treg ratio in tumor microenvironment at 5 days after the last immunization (*n*=5, **P<0.05). Results are representative of three independent experiments. (**b**) The serum samples in all groups were analyzed by mouse cytokine/chemokine CBA include Th1, Th2 cytokines and chemokine. IFN-γ, TNF-α, IL-2, IL-12 were significantly upregulated as shown (*n*=5, **P<0.05). Results are representative of three independent experiments.

To better understand the mechanism of anti-tumor effect in combined vaccine, we collected serum, spleen, lymph node and tumor samples in all groups at 1, 3, 5 and 7 days following the last immunization. The spleen, lymph node and tumor samples were analyzed for DC, MDSC, MΦ, Teff/Treg changes by FACS. The results showed that CD11c^+^CD86^+^ DC and CD11b^+^F4/80^+^MΦ of the combined group in spleen and lymph node slightly increased at 5 days after vaccination. The expression of MHC-II on the surface of DC and MΦ also significantly upregulated, promoting antigen presentation, CD11b^+^Gr-1^+^MDSC decreased (Supplementary Figure 2) compared with control. In spleen, lymph node, tumor tissue, the Teff/Treg ratio of the combined group was all increased at 5 days, especially in tumor tissues the ratio was most significantly increased (*P*<0.01; Figure 5a), The state of immunosuppressive microenvironment was relieved to induce the specific antitumor immune responses. The serum samples in all groups were analyzed by mouse cytokine/chemokine CBA include Th1, Th2 cytokines and chemokines. Several Th1 cytokines (IFN-γ, TNF-α, IL-2 and IL-12) in combined group were significantly up regulated (Figure 5b) compared with control. Th2 cytokine such as IL-4 decreased, and IL-10 did not change obviously (Supplementary Figure 3). Chemokines such as MCP-1, CCL5 increased slightly (Supplementary Figure 3). The concentrations of GM-CSF and PD-1 antibodies *in vivo* were showed in Table 1. These data suggested that the combined vaccine co-expression PD-1 antibody and GM-CSF effectively changed the immunosuppressive state in tumor microenvironment by upregulating the ratio of Teff/Treg and promoting Th1 cytokine secretion.

### The antitumor immune response in the combined vaccine primarily depends on Th1 immune response

**Figure 6:**
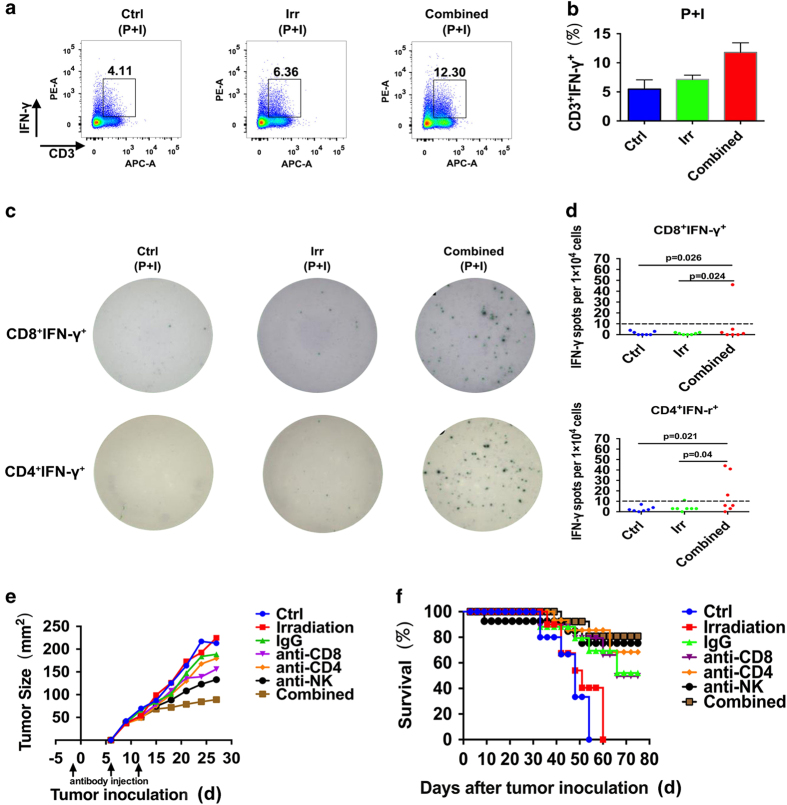
Antitumor mechanism of tumor cell vaccine *in vivo.* (**a**) The percentage of CD3^+^IFN-γ^+^ T cells was analyzed by CD3-APC and IFN-γ-PE antibodies after immunization. (**b**) Statistical chart of flow cytometry result. (**c**) The enzyme-linked immune spot diagram of CD4^+^IFN-γ^+^ and CD8^+^IFN-γ^+^ T cells. (*P*<0.05, *n*=7) (**d**) Splenic cells were collected and plated (100 μl per well) in triplicate 1×10^5^ cells per ml, then were incubated in ELISPOT plates in the presence of 0.5 μg ml^−1^ ionomycin mixed with 50 ng ml^−1^ PMA for 72 h The activity of IFN-γ-secreting cells was measured by the counting spots formed by IFN-γ secreted from individual cells and designated as spot forming cells (SFCs). Statistical figures of ELISPOT result as shown. (**e**) Tumor size of blocking animal experiment. Each mouse was injected with 100 μg anti-CD8, anti-CD4 and anti-NK blocking antibody through tail vein in 1 day before inoculation and 6 days, 13days after inoculation (*n*=6). Data are representative of three independent experiments (**f**) Kaplan–Meier survival analysis of blocking experiments *in vivo* (*n*=8). Results are representative of three independent experiments.

For further obtaining more insight into the cellular mechanisms of Th1 cytokine-mediated inhibition of tumor growth, we analyzed the percentage of CD3^+^IFN-γ^+^ in spleen between the combined group and control by FACS (Figure 6a). The results showed a great difference between these two groups (*P*<0.05; Figure 6b). To compare the difference of IFN-γ secretion in CD4^+^IFN-γ^+^ and CD8^+^IFN-γ^+^ T cells, we confirmed that the ability of cytokine secretion of CD4^+^IFN-γ^+^ T cells and CD8^+^IFN-γ^+^ T cells is much stronger in the combined group than the control group by ELISPOT (Figure 6c). The results showed significant difference in CD4^+^IFN-γ^+^ (*P*=0.021) and CD8^+^IFN-γ^+^ T cells (*P*=0.026; Figure 6d). We also depleted CD4 or CD8 T lymphocytes or NK cells *in vivo* through injection of the corresponding blocking antibodies. Mice treated with mAbs against CD4 or CD8 T cell failed to abrogate the antitumor activity (*P*>0.05). In contrast, depletion of NK (*P*=0.018) or injection of an isotype control rat immunoglobulin-G (IgG) still showed strong antitumor activity (Figure 6e) and significantly prolonged the survival (Figure 6f) compared with the control group. Moreover, immunofluorescence analysis in tumor tissue treated with combined vaccine were extensively infiltrated with higher numbers of CD4^+^T, CD8^+^T immune cells compared with the control (Supplementary Figure 4), immune cell infiltration was observed not only around, but also inside the remaining tumor tissues. These findings suggested that combined vaccine enhanced proliferation and infiltration of CD4^+^ T, CD8^+^ T cells and especially CD4^+^ INF-γ^+^ T cells.

## Discussion

Interactions between the immune system and cancers are governed by a complex network of biological pathways. Despite expectations that the immune system should automatically reject cancer cells as ‘foreign’, based on their unique and often extensive mutational profiles, the overriding natural balance between the immune system and cancer is tolerance, in which cancer cells are seen as ‘self’. Tolerance is maintained by multiple mechanisms, including regulatory immune cells, immunosuppressive cytokines and chemokines, and so called ‘immune checkpoint’ pathways that downmodulate immune functions. PD-1 is a dominant immune checkpoint protein in the tumor microenvironment. Its normal function in controlling immune homeostasis is induced in cancer cells to evade immune attack.^25,26^ Monoclonal antibodies that block this immune checkpoint have emerged as powerful weapons in the oncological armamentarium. Durable objective responses following PD-1 blockade therapy in patients with advanced melanoma (31–44%),^27–31^ Non-small-cell lung cancer (19–20%),^32–34^ Renal cell carcinoma (22–25%),^35,36^ accompanied by extended overall survival compared with conventional therapies. The first immune checkpoint inhibitor ipilimumab, which gained US Food and Drug Administration (FDA) approval in 2011, and two antibodies against PD-1 (pembrolizumab and nivolumab), a dominant immune checkpoint protein were subsequently approved by FDA in 2014.

Although PD-1 blockade for cancer immunotherapy has achieved great successes in melanoma, and will also be approved over the next several years for treatment lung cancer, kidney cancer, bladder cancer, prostate cancer and many other tumor types.it also faces with many problems: (1) The cost for PD-1 antibody therapy is too high. (2) It also can bring systemic side effects and lead to disorders in immune systems. (3) The optimized regimen for PD-1 blockade therapy is still controversial. To solve these problems, in our research we successfully designed and prepared a novel cancer cell vaccine that can simultaneously secret anti-PD-1 mAbs and GM-CSF locally and might allow higher concentrations at the tumor area while reducing systemic side effects. We verified the antitumor effects in CT26 with strong immunogenicity^23^ and B16-F10 with low immunogenicity.^37^ Compared with the control, the combined vaccine can induce strong special anti-tumor effects and extend the survival in both colon cancer and melanoma models. A dominant mechanism that is relevant to anti-PD-1 antibodies response is PD-L1 expression in tissues. PD-L1 is normally expressed by a subset of macrophages and can be induced on activated lymphocytes (T, B and NK cells), endothelial cells and non-malignant cell types in an inflammatory microenvironment, as part of a physiological process to down-modulate ongoing host immune responses in peripheral tissues.^14,38,39^ However, tumor cells and associated stromal cells can also express this checkpoint protein, thereby turning off Teff cells.^14^ Certain cancers, such as melanoma, breast cancer and renal cell carcinoma, frequently express PD-L1 on the surface of tumor cells as well as infiltrating immune cells.^12,40–43^ In contrast, in other tumors such as colorectal carcinoma and gastric carcinoma, PD-L1 is observed almost exclusively on tumor-infiltrating immune cells and is only rarely expressed on tumor cells themselves.^44–46^ In our study, we also analyzed PD-L1 expression in colon tumor and melanoma cell lines by FACS and observed PD-L1 expression in human tissue microarray including melanoma, lung cancer, colon cancer and liver cancer. Immunohistochemistry analysis revealed that ~80% of PD-L1 expression on tumor tissue and PD-1 expression on tumor infiltrating lymphocyte were positive. This may predict relationship between intratumoural PD-L1 expression and PD-1 blockade therapy response.

The complexities of the interaction between cancer and the host immune system are still unclarified. Different components of the immune system can either promote or inhibit tumor growth. The potential for the adaptive immune system, in particular CD8^+^ cytotoxic T lymphocytes, to control or eradicate tumors has been shown in laboratory models. In a study of human colorectal carcinoma specimens detailing the relationship between T-cell densities at the invasive tumor margin and those in the center of the tumor, high densities of CD45R^+^CD3^+^CD8^+^granzyme^+^ T cells (antigen-specific cytolytic Teff cells) were associated with a lower possibility of tumor relapse and improved overall survival.^47^ To further investigate the antitumor mechanism, we analyzed DC, MDSC, MΦ, Teff/Treg changes in the spleen, lymph node and tumor samples by FACS. The results showed that CD11c^+^CD86^+^ DC and CD11b^+^F4/80^+^MΦ of the combined group in spleen and lymph node slightly increased. The expression of MHC-II on the surface of DC and MΦ also significantly upregulated, promoting antigen presentation. In spleen, lymph node, tumor tissue, the Teff/Treg ratio of the combined group was all increased, especially in tumor tissues the ratio was most significantly increased, interestingly CD11b^+^Gr-1^+^MDSC in tumor decreased. We inferred that the possible mechanism caused MDSC apoptosis through Fas/FasL pathway.^48^ TNF-α is a pleiotropic inflammatory factor that can promote inflammation and induce proliferation and apoptosis process.^49^ *In vitro,* we also found TNF-α was significantly upregulated in serum samples. Taken together, the ratio of Teff/Treg in tumor microenvironment is beneficial for relieving the state of immunosuppressive microenvironment and contributes to induction of the specific antitumor immune responses.

Cytokines can mediate the development of inflammation and involved in proliferation, differentiation and chemotaxis. Th1 cytokines can promote intracellular inflammation and inflammatory cytokine production. Th2 cytokines can inhibit inflammatory response and promote B cells to produce antibodies.^37^ Furthermore, we also analyzed serum samples in all groups using mouse cytokine/chemokine CBA, including Th1, Th2 cytokines and chemokines. Several Th1 cytokines (GM-CSF, IFN-γ, TNF-α, IL-2 and IL-12) were significantly upregulated and Th2 cytokines, such as IL-4, decreased, whereas IL-10 did not have significant change. Chemokines, such as MCP-1 and CCL5, increased slightly. To compare the difference of IFN-γ secretion in CD4^+^IFN-γ^+^ and CD8^+^IFN-γ^+^ T cells, we confirmed that the ability of cytokine secretion of CD4^+^IFN-γ^+^ and CD8^+^IFN-γ^+^ T cells were much stronger in the combined group than the control group by ELISPOT. Moreover, immunofluorescence analysis in tumor tissue treated with the combined vaccine was extensively infiltrated with higher numbers of CD4^+^ and CD8^+^T immune cells compared with the control, especially CD4^+^ T cells.

In conclusion, we proved antitumor effect of the novel cancer cell vaccine in mouse colon cancer and melanoma model. The results showed that the cancer cell vaccine secreting PD-1 neutralizing antibodies and GM-CSF induced remarkable antitumor immune effect either in therapeutic or adoptive animal experiments. The vaccine could promote DC and MΦ cells and the ratio of Teff/Treg in the tumor microenvironment. The state of immunosuppressive microenvironment was relieved, hence beneficial for developing stronger specific antitumor immune response. The vaccine can also induce production of Th1 cytokines and expression of certain relevant chemokines, which could promote the recruitment, proliferation and infiltration of immune cells in feedback regulation and greatly enhance antitumor effects. The results of ELISPOT and blocking experiments *in vivo* further substantiated that the antitumor immune response is acquired by CD4 and CD8 T immune responses, mainly dependent on CD4 Th1 immune response.

## Figures and Tables

**Table 1:** The concentration of GM-CSF and anti-PD-1 antibodies *in vivo*

	*Ctrl*	*Irr*	*mGM-CSF*	*αPD-1*	*mGM-CSF+αPD-1*
mGM-CSF (pg ml^−1^)	27.61	26.36	33.14	27.43	91.18
αPD-1 (ng ml^−1^)	17.69	18.89	19.89	23.20	26.50
