# Author Correction: A novel cancer vaccine with the ability to simultaneously produce anti-PD-1 antibody and GM-CSF in cancer cells and enhance Th1-biased antitumor immunity

**DOI:** 10.1038/s41392-019-0065-6

**Published:** 2019-08-28

**Authors:** Hongwei Tian, Gang Shi, Qin Wang, Yiming Li, Qianmei Yang, Chunlei Li, Guoyou Yang, Min Wu, Qian Xie, Shuang Zhang, Yang Yang, Rong Xiang, Dechao Yu, Yuquan Wei, Hongxin Deng

**Affiliations:** 1 State Key Laboratory of Biotherapy/Collaborative Innovation Center for Biotherapy, West China Hospital, Sichuan University, Chengdu, Sichuan China; 2 Department of Biomedical Sciences, University of North Dakota, Grand Forks, North Dakota, USA; 3 Department of General Medicine, West China Hospital, Sichuan University, Chengdu, Sichuan China; 4 Department of Immunology, Nankai University School of Medicine, Tianjin, China

**Correction to:** *Signal Transduction and Targeted Therapy* (2016)1:16025 10.1038/sigtrans.2016.25, published online 18 November 2016

Since the publication of this research Article, we retrospected the paper and noticed two inadvertent mistakes that need to be corrected immediately. We have checked the original data and repeated the experiments; the correct data are provided in this Corrigendum as follows. The key findings of the article are not affected by these corrections.

1. In Fig. 1a, after repeating the experiment, we found that the PD-L1 expression in CT26 and B16-F10 cell lines are different from those before. We checked the original data and confirmed that there is something wrong. Thus, the original figure should be replaced as shown here.
  Therefore the sentence in the results about Fig. 1a: “The results showed that PD-L1 percentage is CT26 (26.65%) and B16-F10 (79.57%)”, should be changed to “The results showed that PD-L1 percentage is CT26 (81.7%) and B16-F10 (44.9%)”. However, immunohistochemistry analysis revealed approximately the same percentage of PD-L1 expression in clinical tissue samples, including colon cancer and melanoma. We think that the difference in expression levels of PD-L1 in tumor cell lines and clinical samples need to be investigated further.
2. In Fig. 2b, the same pictures showed the proliferation of CD8^+^PD-1^+^ TIL treated with supernatant from the C26-anti-PD-1 or C26-GM-CSF+anti-PD-1 vaccines, which is an inadvertent mistake. After checking the original data, we feel the figure should be as shown here.

**Fig. 1a:**
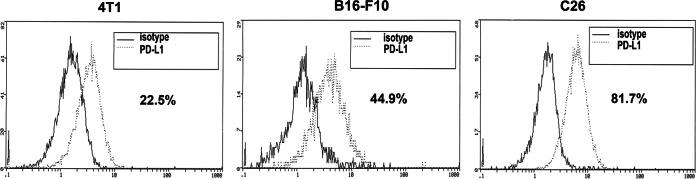
Expression of PD-L1 and PD-1 in mouse tumor cells and human tissues. (**a**) PD-L1 expression on the surface of CT26 and B16-F10 tumor cells was detected by FACS. The black and red line represents isotype and PD-L1, respectively

**Fig. 2b:**
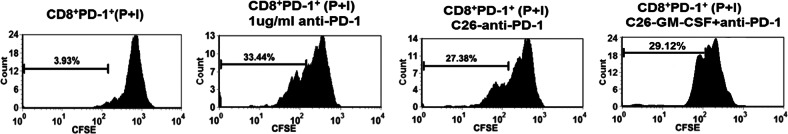
CD8^+^PD-1^−^ and CD8^+^PD-1^+^ TIL in CT26 tumor were sorted and labeled with CFSE in vitro, then treated with supernatant from the CT-26-anti-PD-1 vaccine. The PD-1 antibody was used as a positive control at the concentration of 1 μg·ml^−1^. After PMA and Ionomycin stimulation for 72 h, the proliferation was detected by FACS

We apologize for this omission.

