# Supplementary material for: A novel cancer vaccine with the ability to simultaneously produce anti-PD-1 antibody and GM-CSF in cancer cells and enhance Th1-biased antitumor immunity
Source: Signal Transduct Target Ther. 2016 Nov 18;1:16025–. doi: 10.1038/sigtrans.2016.25 (PMC5661645; doi:10.1038/sigtrans.2016.25)
Supplement: Supplementary Information [file sigtrans201625-s1.pdf]

Supplementary Fig. 1

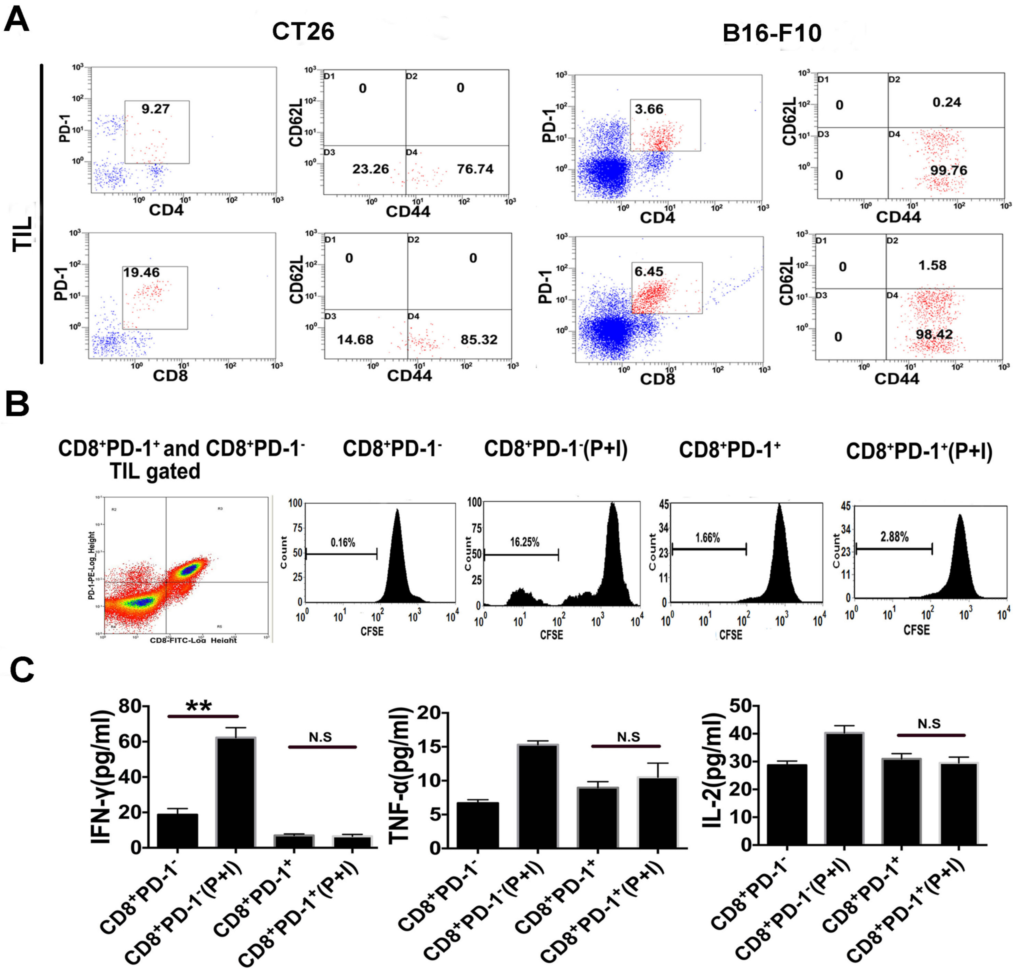

Supplementary S1 Characterization of PD-1^{+}TIL in tumor microenvironment. (A) CT26 and B16-F10 tumor tissue suspension were prepared and stained with CD45-APC, CD4-FITC, CD8-PE/Cy7, PD-1-PE, CD44-PE/Cy5.5 and CD62L-APC/Cy7 flow antibodies to analyze the percentage of CD4^{+}PD-1^{+} and CD8^{+}PD-1^{+} TIL. The results showed PD-1 were high positively expressed on CD4^{+}/CD8^{+}CD44^{+}CD62L^{-} T effector in CT26 and B16-F10. (B) CD8^{+}PD-1^{-} and CD8^{+}PD-1^{+} TIL were separately sorted in CT26 tumor and labeled it with CFSE in vitro. After PMA and Ionomycin stimulation for 72 hours, the proliferation was detected by FACS and. (C) The supernatant after ELISA

collected 72 hours in all groups to detect IFN-γ, TNF-α and IL-2 secretion. Data are means± SD (n=3) and are representative of three experiments (**, $P < 0.05$).

Supplementary Fig. 2

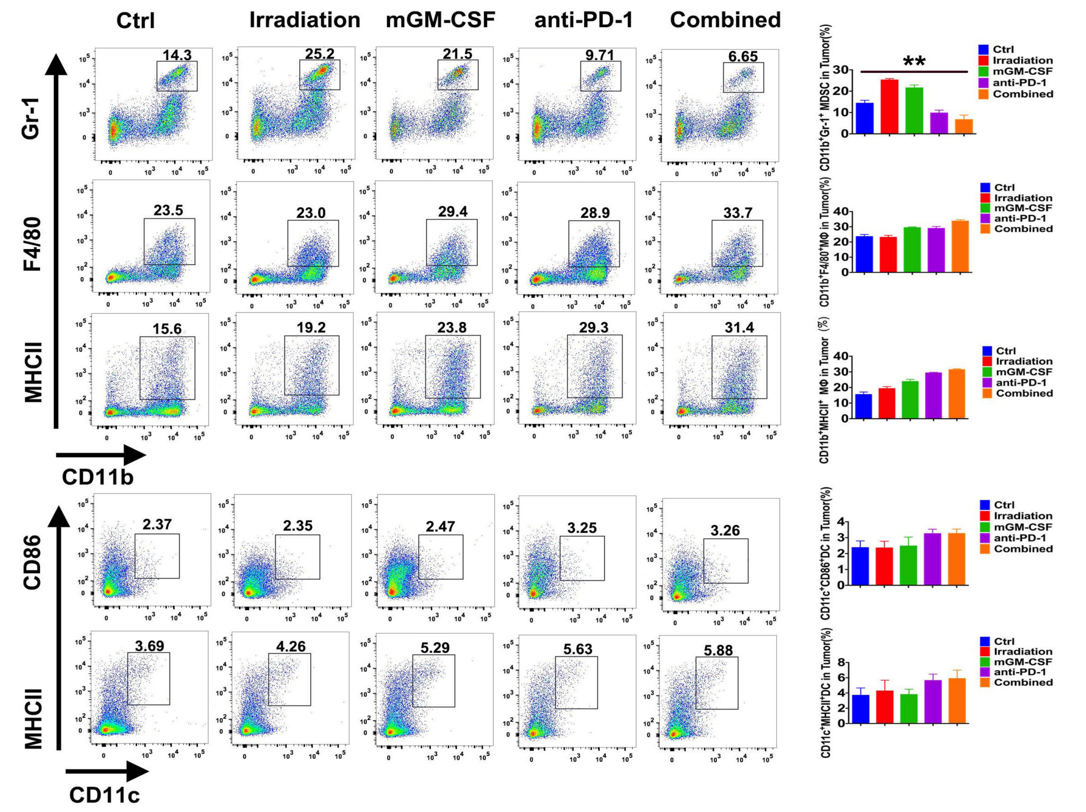

Supplementary S2 Changes of MDSC、DC、MΦ in tumor microenvironment.

Supplementary Fig. 3

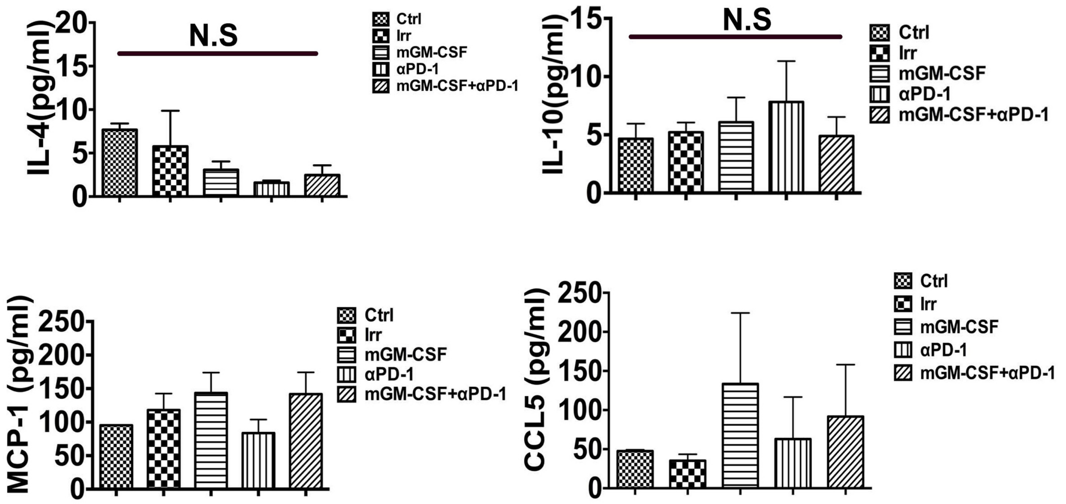

Supplementary S3 Expression of IL-4, IL-10 cytokine and MCP-1, CCL5 chemokine in serum.

Supplementary Fig. 4

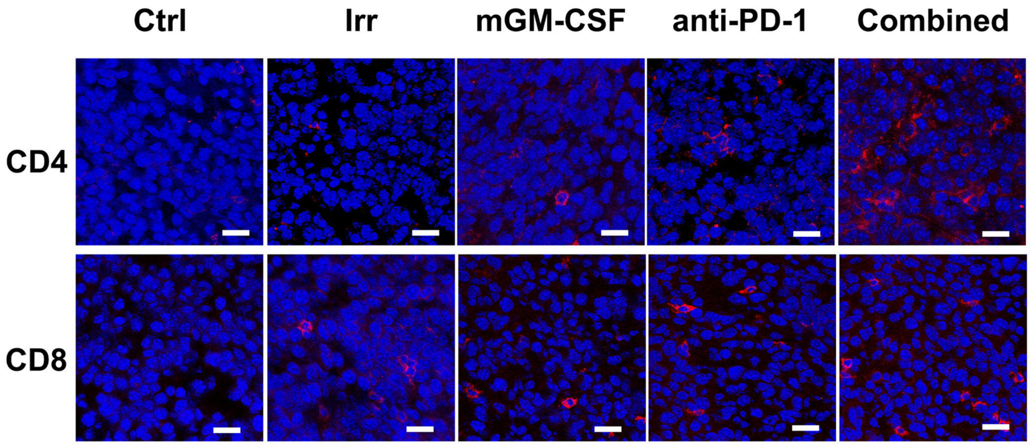

Supplementary S4 Infiltration of lymphocyte in tumor was analyzed by immunofluorescence. Red positive signals represent $CD4^+$, $CD8^+$ T lymphocytes. Areas on the figures were taken by confocal under 200×.
